# Supplementary material for: Carotenoid metabolism strengthens the link between feather coloration and individual quality
Source: Nat Commun. 2018 Jan 8;9:73. doi: 10.1038/s41467-017-02649-z (PMC5758789; doi:10.1038/s41467-017-02649-z)
Supplement: Supplementary file 1 — Supplementary Information [file 41467_2017_2649_MOESM1_ESM.pdf]

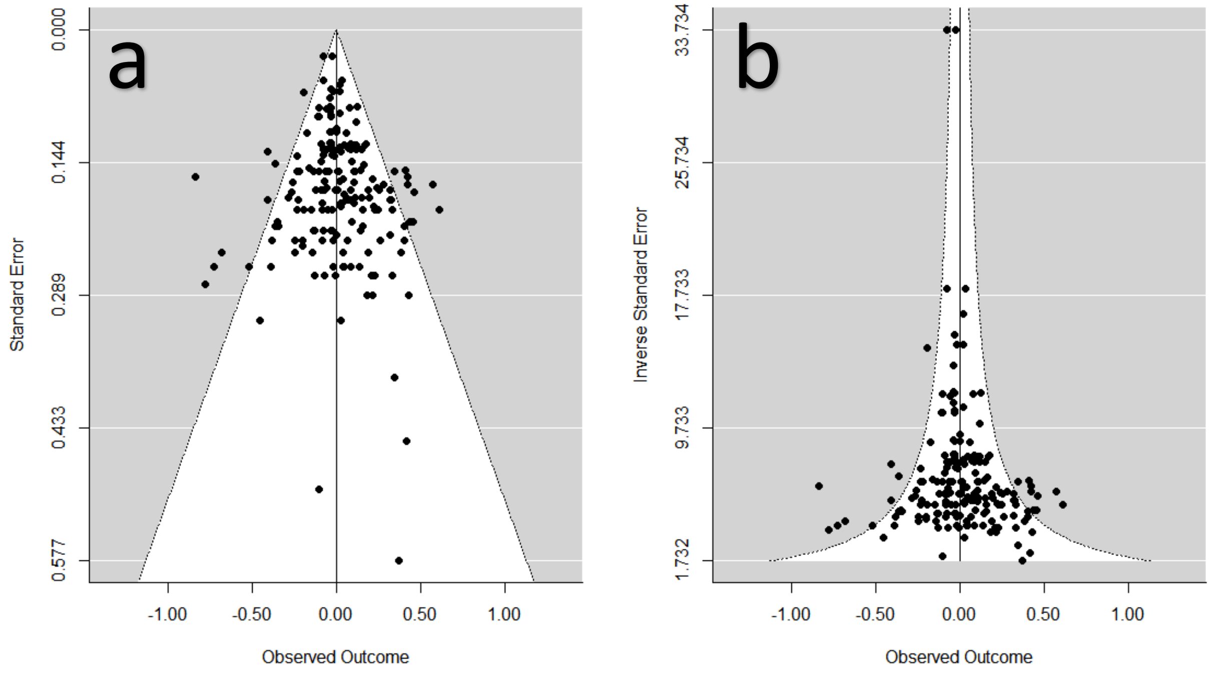

Supplementary Figure 1. Funnel plots of meta-analytic residuals. Effect size (Zr) plotted against (**a)** standard error and **(b)** precision (inverse of the standard error) n = 191 effect sizes.

PRISMA 2009 Flow Diagram

Corresponding Article Title: Carotenoid metabolism strengthens the link between feather coloration and individual quality

Search terms:

(“carotenoid”, “color”, “condition”, “signal”, “feather”, “quality” and all possible alternative spellings and combinations)

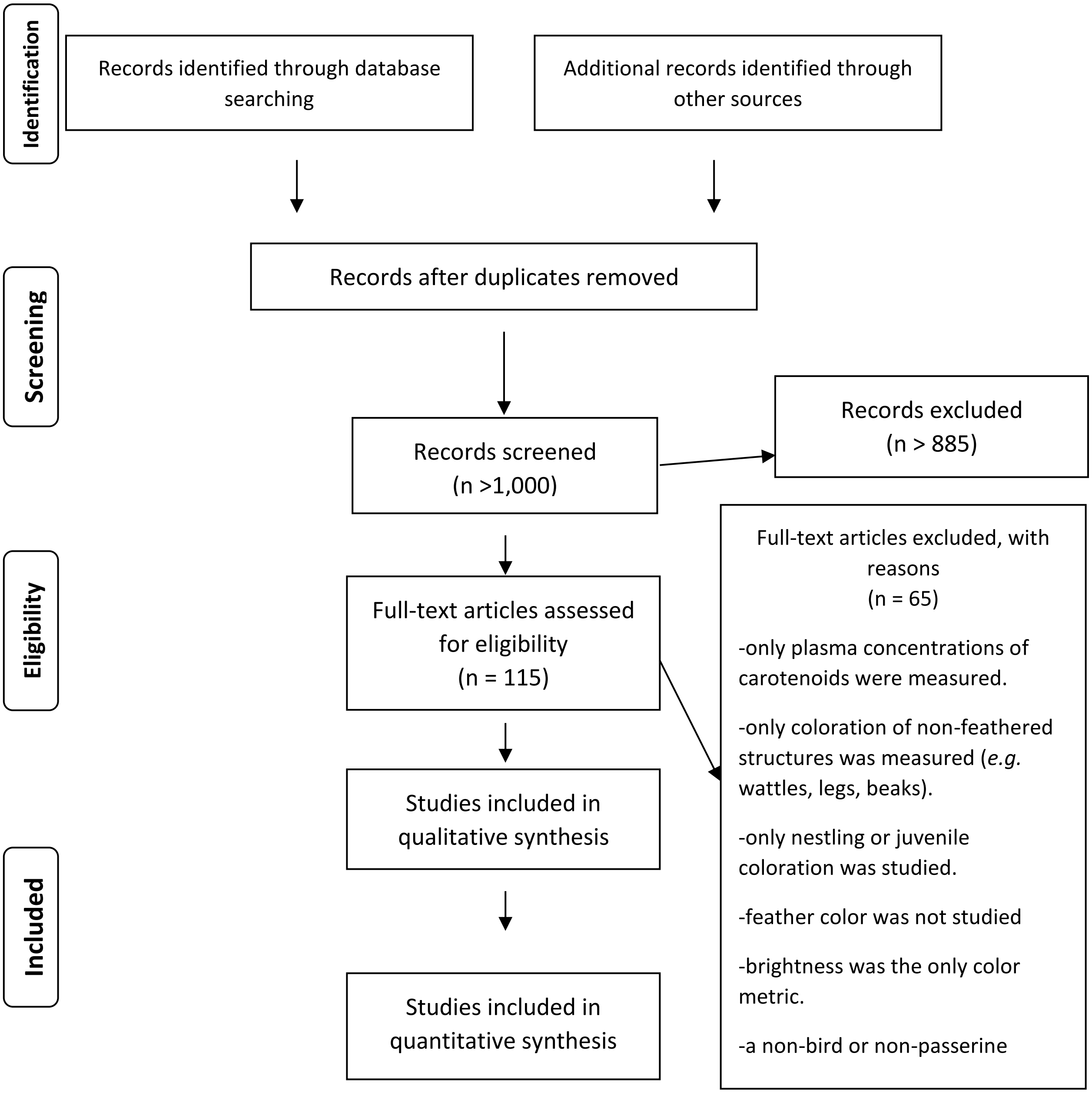

Supplementary Figure 2. PRISMA flow diagram

Supplementary Table 1. Model assuming effect sizes are not correlated

Analysis	*k*	*m*	Mean (*Zr*)	Lower CI (2.5%)	Upper CI (97.5%)	I^2(%)
Overall	191	19	0.178	-0.038	0.414	64.67
Carotenoid Type:						61.26
Converted	92	12	**0.255**	**0.030**	**0.506**	
Dietary	99	7	0.077	-0.134	0.318	
Category (combined)						66.27
Condition	35	12	0.069	-0.148	0.328	
Immune and Oxidative	42	10	0.142	-0.077	0.406	
Parasite Resistance	49	10	0.237	-0.020	0.467	
Reproductive and Parental Quality	65	9	0.225	-0.020	0.451	
Category (Converted)						57.91
Condition	18	8	0.104	-0.161	0.390	
Immune and Oxidative	21	6	0.133	-0.162	0.414	
Parasite Resistance	30	8	**0.423**	**0.135**	**0.688**	
Reproductive and Parental Quality	23	3	**0.368**	**0.067**	**0.677**	
Category (Dietary)						
Condition	17	4	0.106	-0.163	0.385	
Immune Function	21	4	0.129	-0.154	0.384	
Parasite Resistance	19	3	0.029	-0.256	0.276	
Reproductive and Parental Quality	42	6	0.080	-0.147	0.365	

k = number of effect sizes, *m* = number of species. Category (Converted) and Category (Dietary) represent estimates from a meta-analytic model with an interaction between life history trait category and type of carotenoid as predictor variables. Effect sizes in bold are considered to be statistically significantly different from 0, as the 95% credible interval did not overlap 0.

Supplementary Table 2. Model with measures of oxidative physiology split from immune category

Analysis	*k*	*m*	Mean (*Zr)*	Lower CI (2.5%)	Upper CI (97.5%)
Category (Converted)					
Condition	18	8	0.077	-0.238	0.347
Immune	20	6	0.095	-0.205	0.42
Oxidative	1	1	0.107	-0.515	0.724
Parasite	30	8	**0.438**	**0.173**	**0.751**
Reproductive and Parental Quality	23	3	**0.333**	**0.011**	**0.647**
Category (Dietary)					
Condition	17	4	0.101	-0.196	0.397
Immune	12	4	0.045	-0.286	0.359
Oxidative	9	2	0.222	-0.116	0.592
Parasite	19	3	0.003	-0.256	0.298
Reproductive and Parental Quality	42	6	0.089	-0.211	0.358

k = number of effect sizes*, m* = number of species*.* Category (Converted) and Category (Dietary) represent estimates from a meta-analytic model with an interaction between life history trait category and type of carotenoid as predictor variables. Effect sizes in bold are considered to be statistically significantly different from 0, as the 95% credible interval did not overlap 0.

Supplementary Table 3. Model with parasite measures lumped with immune category, but oxidative measures in a separate category

	k	*m*	Mean (*Zr)*	Lower CI (2.5%)	Upper CI (97.5%)
Category (Converted)					
Condition	18	8	0.077	-0.138	0.341
Immune	50	11	**0.318**	**0.094**	**0.560**
Oxidative	1	1	0.082	-0.534	0.752
Reproductive and Parental Quality	23	3	**0.354**	**0.092**	**0.661**
Category (Dietary)					
Condition	17	4	0.097	-0.195	0.339
Immune	31	5	0.020	-0.196	0.268
Oxidative	9	2	0.236	-0.107	0.526
Reproductive and Parental Quality	42	6	0.103	-0.115	0.360

k = number of effect sizes*, m* = number of species. Category (Converted) and Category (Dietary) represent estimates from a meta-analytic model with an interaction between life history trait category and type of carotenoid as predictor variables. Effect sizes in bold are considered to be statistically significantly different from 0, as the 95% credible interval did not overlap 0.

Supplementary Table 4. Types of carotenoids in feathers of species included in the meta-analyses.

Species	Color	Carotenoid Type	Reference
American Goldfinch	Yellow	Converted	[1]
Blue Tit	Yellow	Dietary	[2]
Cirl Bunting	Yellow	Dietary *	[2]
Common Redpoll	Red	Converted	[2,3]
Common Rosefinch	Red	Converted	[4]
Common Yellowthroat	Yellow	Dietary	[5]
European Greenfinch	Yellow	Converted	[2]
European Serin	Yellow	Converted	[6]
Golden-Collared Manakin	Yellow	Dietary *	[7]
Golden-crowned Kinglet	Yellow	Converted	[8]
Great Tit	Yellow	Dietary	[9]
House Finch	Red	Converted	[9]
Kentucky Warbler	Yellow	Dietary *	[5]
Linnet	Red	Converted	[4]
Northern Cardinal	Red	Converted	[3]
Red Fody	Red	Converted	[9]
Red-winged Blackbird	Red	Converted	[10]
Southern Red Bishop	Red	Converted	[9]
Yellowhammer	Yellow	Dietary	[9]

* No published reports for this species, carotenoid type estimated by comparison to sister-species of the same genus that displays the same feather color.

Supplementary Note 1. Carotenoid structure, nomenclature, and metabolic transformations.

Carotenoids used for coloration in most animals are C_{40} tetraterpenoids. They consist of a central polyene chain — a system of conjugated carbon bonds that comprises most of the 'chromophore' (*i.e.* part of the molecule that reflects light) — with ionone rings at either end. These hydrocarbon carotenoids are called 'carotenes' whose specific names are derived from the types of ionone end rings present. β-carotene (β, β-carotene) contains two β-ionone rings, while α-carotene (β, ε -carotene) contains one β-ionone and one ε-ionone ring. Carotenoids containing at least one unmodified β-ionone ring can be cleaved by most animals to yield retinal and thus have pro-vitamin A potential. Modifications to the end rings though oxidation reactions determine the function and color of the carotenoid by increasing its polarity and/or chromophore length. The addition of conjugated double bonds lengthens the chromophore and increases peak light absorption from shorter to longer wavelengths, causing a shift from yellow towards red color (a bathochromic shift).

Carotenes can be modified by the addition of oxygen (as hydroxyl or ketone functional groups) to carbons 3 or 4 of the ionone end rings through oxygenation or dehydrogenation reactions. These oxygenated carotenoids are broadly known as 'xanthophylls'. Specific xanthophyll names are determined by the presence of either one or more hydroxyl groups (hydroxy-carotenoids) and/or ketone groups (keto-carotenoids) to the ionone rings. Zeaxanthin (3,3'-dihydroxy-β-carotene) and lutein (3,3'-dihydroxy- α -carotene) are common hydroxy-carotenoids that are abundant in the diet of many herbivorous and insectivorous animals. In contrast, keto-carotenoids such as echinenone (4-keto-β-carotene), canthaxanthin (4,4'-diketo-β-carotene), and astaxanthin (3,3'-dihydroxy-4,4'diketo -β-carotene) are mostly absent from animal diets. Instead, keto-carotenoids are produced either through ketolation of hydroxy-carotenoids or through hydroxylation and ketolation of carotenes and are responsible for most of the vibrant red hues of animal integuments. However, not all keto-carotenoids yield red coloration; 'canary xanthophylls'—ketolated products of lutein and zeaxanthin that are derived from dehydrogenation of the existing hydroxyl groups —produce a rich yellow color used by some songbirds as feather pigments. The mechanism by which the ketone is formed includes a change from β-ionone rings to ε-ionone rings which shortens the conjugated system (shortens the chromophore) causing canary xanthophylls appear yellow and not red.

Despite the prevalence and importance of carotenoids in animals, the genetic architecture and physiological mechanisms involved in carotenoid metabolism have only recently been identified. In 2016, two independent lab groups characterized the genetic basis for red bill and red feather coloration, dubbed the *redness* gene. This gene encodes a cytochrome P450 oxidoreductase *CYP2J19* that catalyzes the oxidative transformation of dietary carotenoids to hydroxy- or keto-carotenoids Identification of the particular mechanisms and cellular locations involved in hydroxylation and ketolation of carotenoids in animals is currently underway.

Supplementary References

1. McGraw, K. J., Hill, G. E., Stradi, R. & Parker, R. S. The influence of carotenoid acquisition and utilization on the maintenance of species-typical plumage pigmentation in male American goldfinches (*Carduelis tristis*) and northern cardinals (*Cardinalis cardinalis*). *Physiol. Biochem. Zool.* **74,** 843–852 (2001).

2. Stradi, R. *The Colour of Flight: Carotenoids in Bird Plumage*. (Solei Gruppo Editoriale Informatico, 1998).

3. McGraw, K. J. in *Bird Coloration, Volume 1: Measurements and Mechanisms* (eds. Hill, G. E. & McGraw, K. J.) (Harvard University Press, 2006).

4. Stradi, R., Celentano, G., Boles, M. & Mercato, F. Carotenoids in Bird Plumage: The Pattern in a Series of Red-Pigmented Carduelinae. *Comp. Biochem. Physiol. Part B Biochem. Mol. Biol.* **117,** 85–91 (1997).

5. McGraw, K. ., Beebee, M. ., Hill, G. . & Parker, R. . Lutein-based plumage coloration in songbirds is a consequence of selective pigment incorporation into feathers. *Comp. Biochem. Physiol. Part B Biochem. Mol. Biol.* **135,** 689–696 (2003).

6. Stradi, R., Celentano, G., Rossi, E., Rovati, G. & Pastore, M. Carotenoids in bird plumage-I. The carotenoid pattern in a series of palearctic Carduelinae. *Comp. Biochem. Physiol. -- Part B Biochem.* **110,** 131–143 (1995).

7. Hudon, J., Capparella, A. P. & Brush, A. H. Plumage Pigment Differences in Manakins of the *Pipra Erythrocephala* Superspecies. *Auk* **106,** 34–41 (1989).

8. Chui, C. K. S., McGraw, K. J. & Doucet, S. M. Carotenoid-based plumage coloration in golden-crowned kinglets *Regulus satrapa*: pigment characterization and relationships with migratory timing and condition. *J. Avian Biol.* **42,** 309–322 (2011).

9. Stradi, R. in *Colori in volo: il piumaggio degli uccelli* (eds. Brambilla, L. et al.) 117–146 (Universita degli Studi di Milano, 1999).

10. McGraw, K. J., Wakamatsu, K., Clark, A. B. & Yasukawa, K. Red-winged blackbirds *Agelaius phoeniceus* use carotenoid and melanin pigments to color their epaulets. *J. Avian Biol.* **35,** 543–550 (2004).
