# Supplementary material for: Carotenoid metabolism strengthens the link between feather coloration and individual quality
Source: Nat Commun. 2018 Jan 8;9:73. doi: 10.1038/s41467-017-02649-z (PMC5758789; doi:10.1038/s41467-017-02649-z)
Supplement: Supplementary file 3 — Description of Additional Supplementary Files [file 41467_2017_2649_MOESM3_ESM.pdf]

File Name: Supplementary Data 1
Description: Data used in code to perform meta-analyses.

File Name: Supplementary Data 2
Description: References for studies included in the meta-analyses.

File Name: Supplementary Data 3
Description: R code used to perform meta-analyses.
